# Supplementary figures and images for: The Bank Vole (Clethrionomys glareolus)—Small Animal Model for Hepacivirus Infection
Source: Viruses. 2021 Dec 3;13(12):2421. doi: 10.3390/v13122421 (PMC8708279; doi:10.3390/v13122421)

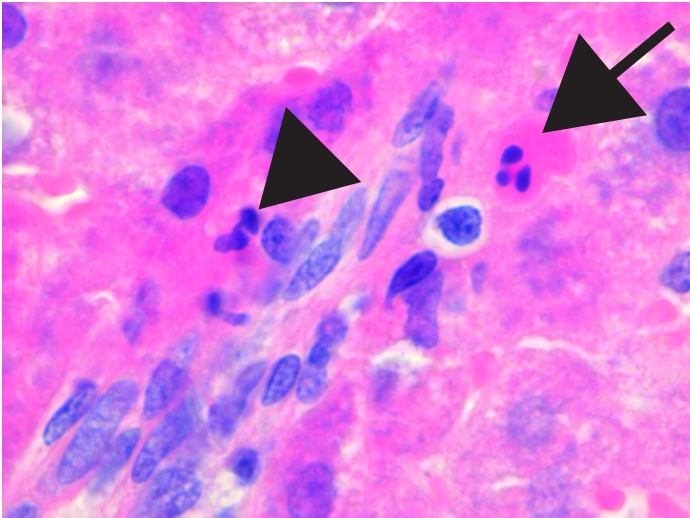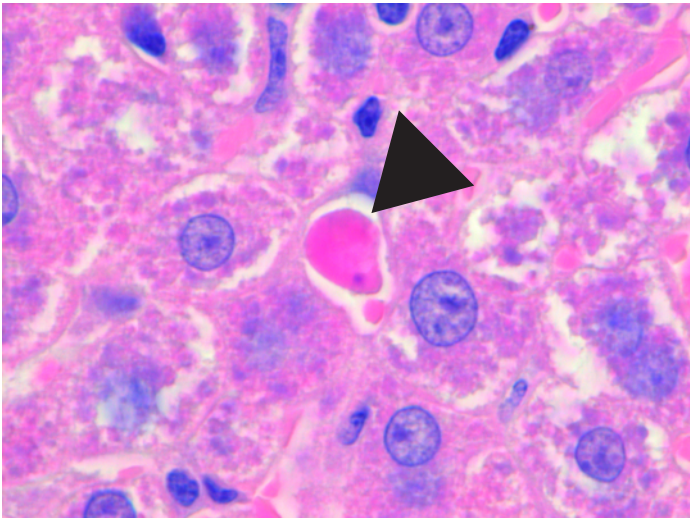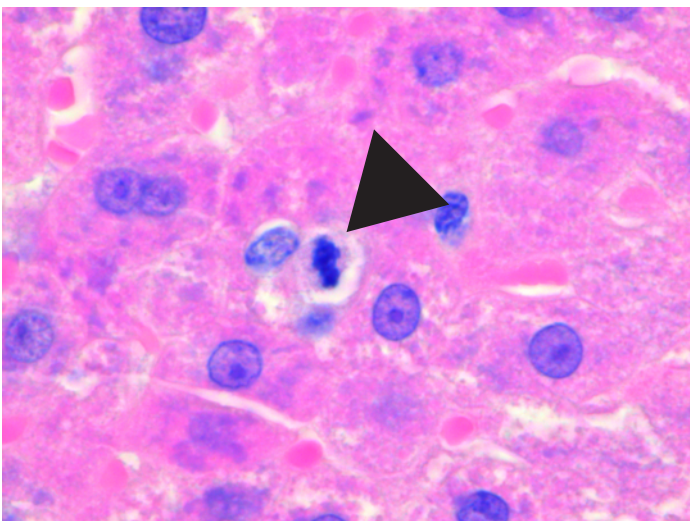

Supplement: Supplementary file 1 [file viruses-13-02421-s001.zip › Suppl Fig 1.pdf]
